# The cost‐effectiveness of progesterone in preventing miscarriages in women with early pregnancy bleeding: an economic evaluation based on the PRISM trial

**DOI:** 10.1111/1471-0528.16068

**Published:** 2020-01-30

**Authors:** CB Okeke Ogwulu, I Goranitis, AJ Devall, V Cheed, ID Gallos, LJ Middleton, HM Harb, HM Williams, A Eapen, JP Daniels, A Ahmed, R Bender‐Atik, K Bhatia, C Bottomley, J Brewin, M Choudhary, S Deb, WC Duncan, AK Ewer, K Hinshaw, T Holland, F Izzat, J Johns, M Lumsden, P Manda, JE Norman, N Nunes, CE Overton, K Kriedt, S Quenby, S Rao, J Ross, A Shahid, M Underwood, N Vaithilingham, L Watkins, C Wykes, AW Horne, D Jurkovic, A Coomarasamy, TE Roberts

**Affiliations:** ^1^ Health Economics Unit College of Medical and Dental Sciences Institute of Applied Health Research University of Birmingham Birmingham UK; ^2^ Health Economics Unit Centre for Health Policy Melbourne School of Population and Global Health The University of Melbourne Melbourne Vic. Australia; ^3^ College of Medical and Dental Sciences Institute of Metabolism and Systems Research University of Birmingham Birmingham UK; ^4^ College of Medical and Dental Sciences Institute of Applied Health Research University of Birmingham Birmingham UK; ^5^ Carver College of Medicine University of Iowa Health Care Iowa City IA USA; ^6^ Faculty of Medicine & Health Sciences Queen's Medical Centre University of Nottingham Nottingham UK; ^7^ Sunderland Royal Hospital City Hospitals Sunderland NHS Foundation Trust Sunderland UK; ^8^ The Miscarriage Association Wakefield UK; ^9^ Burnley General Hospital East Lancashire Hospitals NHS Trust Burnley UK; ^10^ University College Hospital University College London Hospitals NHS Foundation Trust London UK; ^11^ Tommy’s Charity London UK; ^12^ Royal Victoria Infirmary Newcastle upon Tyne Hospitals NHS Foundation Trust Newcastle upon Tyne UK; ^13^ Queen’s Medical Centre Nottingham University Hospitals NHS Trust Nottingham UK; ^14^ MRC Centre for Reproductive Health the Queen's Medical Research Institute University of Edinburgh Edinburgh UK; ^15^ Guy’s and St Thomas’ Hospital Guy's and St Thomas' NHS Foundation Trust London UK; ^16^ University Hospital Coventry University Hospitals Coventry and Warwickshire NHS Trust Coventry UK; ^17^ Kings College Hospital King's College Hospital NHS Foundation Trust London UK; ^18^ Academic Unit of Reproductive and Maternal Medicine University of Glasgow Glasgow UK; ^19^ James Cook University Hospital South Tees Hospitals NHS Foundation Trust Middlesbrough UK; ^20^ Faculty of Health Sciences University of Bristol Bristol UK; ^21^ West Middlesex University Hospital Chelsea and Westminster Hospital NHS Foundation Trust Isleworth UK; ^22^ St Michael's Hospital University Hospitals Bristol NHS Foundation Trust Bristol UK; ^23^ Biomedical Research Unit in Reproductive Health University of Warwick Warwick UK; ^24^ Whiston Hospital St Helen’s and Knowsley Teaching Hospitals NHS Trust Whiston, Prescot UK; ^25^ Whipps Cross Hospital Barts Health NHS Trust Leytonstone, London UK; ^26^ Princess Royal Hospital Shrewsbury and Telford Hospital NHS Trust Apley Telford UK; ^27^ Portsmouth Hospitals NHS Trust Queen Alexandra Hospital Cosham, Portsmouth UK; ^28^ Liverpool Women’s Hospital Liverpool Women’s NHS Foundation Trust Liverpool Women’s Hospital Liverpool UK; ^29^ East Surrey Hospital, Surrey and Sussex Healthcare NHS Trust Redhill UK

**Keywords:** Cost‐effectiveness, economic evaluation, miscarriage, progesterone

## Abstract

**Objectives:**

To assess the cost‐effectiveness of progesterone compared with placebo in preventing pregnancy loss in women with early pregnancy vaginal bleeding.

**Design:**

Economic evaluation alongside a large multi‐centre randomised placebo‐controlled trial.

**Setting:**

Forty‐eight UK NHS early pregnancy units.

**Population:**

Four thousand one hundred and fifty‐three women aged 16–39 years with bleeding in early pregnancy and ultrasound evidence of an intrauterine sac.

**Methods:**

An incremental cost‐effectiveness analysis was performed from National Health Service (NHS) and NHS and Personal Social Services perspectives. Subgroup analyses were carried out on women with one or more and three or more previous miscarriages.

**Main outcome measures:**

Cost per additional live birth at ≥34 weeks of gestation.

**Results:**

Progesterone intervention led to an effect difference of 0.022 (95% CI −0.004 to 0.050) in the trial. The mean cost per woman in the progesterone group was £76 (95% CI −£559 to £711) more than the mean cost in the placebo group. The incremental cost‐effectiveness ratio for progesterone compared with placebo was £3305 per additional live birth*.* For women with at least one previous miscarriage, progesterone was more effective than placebo with an effect difference of 0.055 (95% CI 0.014–0.096) and this was associated with a cost saving of £322 (95% CI −£1318 to £673).

**Conclusions:**

The results suggest that progesterone is associated with a small positive impact and a small additional cost. Both subgroup analyses were more favourable, especially for women who had one or more previous miscarriages. Given available evidence, progesterone is likely to be a cost‐effective intervention, particularly for women with previous miscarriage(s).

**Tweetable abstract:**

Progesterone treatment is likely to be cost‐effective in women with early pregnancy bleeding and a history of miscarriage.

## Tweetable abstract

Progesterone treatment is likely to be cost‐effective in women with early pregnancy bleeding and a history of miscarriage.

## Introduction

Miscarriage is defined as the loss of an unborn baby before the 24th week of pregnancy.^1^ It is the commonest adverse outcome of pregnancy,^2^, ^3^ with 20–25% of pregnancies ending in a miscarriage.^4^ Miscarriage is associated with substantial adverse clinical and psychological impacts on women and their families^5^ and poses a significant economic burden of an estimated £350 million per year to the UK National Health Service (NHS), for the management of miscarriage and complications.^1^, ^6^

Progesterone is a hormone that is naturally secreted by the ovaries and placenta in early pregnancy and is vital to the attainment and maintenance of healthy pregnancies.^4^ Its physiological importance has led clinicians, researchers and patients to consider progesterone supplementation during early pregnancy as a miscarriage prevention strategy, particularly in women at high risk of miscarriage, such as those with a history of recurrent miscarriages or early pregnancy bleeding.^7^

In 2012, the National Institute for Health and Care Excellence (NICE) guidelines on ‘Ectopic Pregnancy and Miscarriage’ called for a large randomised clinical trial to explore the potential role of progesterone in women with early pregnancy bleeding.^6^, ^8^ The PRogesterone In Spontaneous Miscarriage (PRISM) trial was funded by the UK National Institute for Health Research (NIHR) to investigate the effectiveness and cost‐effectiveness of progesterone on pregnancy outcomes in women with first‐trimester vaginal bleeding.

We report the economic evaluation carried out alongside the PRISM trial. The objective of our study is to explore the relative costs and benefits of using progesterone compared with placebo to prevent miscarriage and achieve a live birth at or beyond 34 weeks of pregnancy.

## Methods

### Trial design and participants

The PRISM trial is a multi‐centre, randomised, double‐blind, placebo‐controlled trial. Detailed information about the trial design and findings is published elsewhere.^7^ Briefly, between May 2015 and July 2017, 4153 women with early pregnancy bleeding and an ultrasonography‐confirmed intrauterine sac were recruited from 48 hospitals across the UK.

Inclusion and exclusion criteria are detailed elsewhere^7^ and are available in the supporting information (see Supplementary material, Appendix [Link], [Link], [Link]). Written informed consent was provided by all trial participants. Ethical approval was obtained from the South Central–Oxford C Research Ethics Committee (REC ref: 14/SC/1345) and the UK Health Research Authority. This study is an economic evaluation that used data collected from a Clinical Trial, hence patients were not involved in the development of the study. The study was funded by the UK NIHR Health Technology Assessment programme (project number HTA 12/167/26).

### Intervention

Women were randomised to either progesterone (400 mg, i.e. two Utrogestan 200 mg pessaries, twice daily) or identical placebo pessaries at a 1 : 1 ratio. The pessaries were administered until 16 full weeks of pregnancy or less if a termination of pregnancy was confirmed before 16 weeks of gestation.

### Outcomes

Outcomes were assessed at three points: 11–14 weeks of gestation, end of pregnancy and 28 days after delivery.^7^ The primary outcome for the cost‐effectiveness analysis (CEA) was live birth at ≥34 completed weeks of gestation. An additional outcome of the PRISM trial was neonatal survival at 28 days postpartum and we explored this as a secondary outcome in the economic evaluation.

### Resource use and costs

Resource use data were collected prospectively using researcher‐recorded data collection forms and health services self‐completed questionnaires (at registration and trial end). Resource use data during antenatal and postnatal periods related to hospital visits, day assessment unit visits, emergency visits and hospital admissions. For the intrapartum period, we collected information on the mode of delivery and pregnancy outcome. Where pregnancy ended as miscarriage, the management was categorised as spontaneous resolution, medical management, or surgical management. The immediate postnatal care resource use included the number of nights of maternal admission to a high dependency unit (HDU) (level 2 care) or intensive therapy unit (ITU) (level 3 care). Neonatal care resource use included the number of nights of the neonate receiving intensive care, high dependency care, or special care. Primary care resource use included contacts with the general practitioner, midwife and social care providers such as social workers. Data were also collected for severe adverse events occurring during the trial.

Unit costs were identified from established national sources and are listed in Table 1.^9^, ^10^, ^11^, ^12^, ^13^ All costs were expressed in UK pounds sterling using 2017/18 as the base price year. Where necessary, costs were inflated using the Hospital and Community Health Services pay and prices index.^14^ The cost of progesterone was £21 for a 21‐pack,^15^ translating to a daily cost of £4 (based on the trial’s dosage of two pessaries twice daily). Ultrasonography costs were not included because these were equally applied to both arms of the trial.

**Table 1:** Unit costs of Resource Items (2017/18 prices)

Resource use items	Unit cost (£)	HRG code	Source*
**Intervention**			
Progesterone (Utrogestan®) 200 mg	4	n/a	BNF^15^
**Antenatal period**			
Antenatal hospital visit (Routine observation)	468	NZ16Z	NHS reference cost^9^
Antenatal DAU (Specialised non‐routine US)	125	NZ22Z	NHS reference cost^9^
Emergency visit (Diagnostic procedures)	118	NZ23Z	NHS reference cost^11^
Inpatient admission <24 hours (Day case management of antenatal disorder)	303	NZ20B	NHS reference cost^9^
Night of patient admission	395		PSSRU^13^
**Delivery mode**			
Unassisted vaginal delivery (no complications)	1840	NZ30C	NHS reference cost^9^
Unassisted vaginal delivery (complications)	2187	NZ30A, NZ30B	NHS reference cost^9^
Instrumental vaginal delivery (no complications)	2302	NZ40C	NHS reference cost^9^
Instrumental vaginal delivery (complications)	2446	NZ40A, NZ40B	NHS reference cost^9^
Elective caesarean section (no complications)	3257	NZ50C	NHS reference cost^9^
Elective caesarean section (complications)	4079	NZ50A, NZ50B	NHS reference cost^9^
Emergency caesarean section (no complications)	4378	NZ51C	NHS reference cost^9^
Emergency caesarean section (complications)	5678	NZ51A, NZ51B	NHS reference cost^9^
Vaginal breech delivery (no complications)	1840	NZ30C	NHS reference cost^9^
Vaginal breech delivery (complications)	2187	NZ30A, NZ30B	NHS reference cost^9^
**Management**			
Spontaneous resolution (Miscarriage without complications)	619	MB08B	NHS reference cost^9^
Surgical management (Miscarriage with complications)	1880	MB08A	NHS reference cost^9^
Medical management (Miscarriage with complications)	1880	MB08A	NHS reference cost^9^
**Postnatal period**			
Admission to HDU (level 2 care)	965	XC06Z	NHS reference cost^9^
Admission to ITU (level 3 care)	1586	XC01Z‐XC05Z	NHS reference cost^9^
Hospital visit	145	n/a	PSSRU^13^
Day assessment unit	125	NZ22Z	NHS reference cost^9^
Emergency visit	98	VB09Z, VB11Z	NHS reference cost^9^
Inpatient admissions (<24 hours)	299	NZ26B	NHS reference cost^9^
Night of inpatient admissions	395	n/a	PSSRU^13^
**Neonatal care**			
Neonatal intensive care	1318	XA01Z	NHS reference cost^9^
Neonatal high dependency care	913	XA02Z	NHS reference cost^9^
Neonatal special care	514	XA03Z‐XA04Z	NHS reference cost^9^
**Primary‐care services (contacts)**			
GP visits	39	n/a	Curtis and Burns^10^
Practice/Community Midwife	30	n/a	Curtis and Burns^12^
Practice nurse visits	9.5	n/a	Curtis and Burns^10^
Psychologist (or counsellor) visits	20	n/a	Curtis and Burns^10^
Health visitor visits	22	n/a	Curtis and Burns^12^
Social worker visits	20	n/a	Curtis and Burns^10^
Number of other community services	21	n/a	Curtis and Burns^10^

DAU, day assessment unit; GP, general practitioner; NHS, National Health Service; PSSRU, Personal Social Services Unit.

All unit costs are inflated to 2017/18 costs using the UK Hospital and Community Health Services pay and prices index.

* Taken from NHS reference costs (2016/17) unless otherwise stated. Where the NHS categories differ from ours, data were extracted from the closest match.

The delivery modes were categorised based on the level of complications,^9^ and weighted averages of the unit costs for the different levels of complications were estimated. As there was no Healthcare Resource Group (HRG) code available for breech delivery, the cost was assumed (in agreement with the clinical team) to be the same as the cost of a normal vaginal delivery. Labour onset costs were not included as these costs are typically incorporated in the delivery mode costs. Published definitions for level 2 care (patient receiving single‐organ support) and level 3 care (patient receiving at least two‐organ support),^16^ were used to define the costs.^9^ No clinically specified severe adverse events were ascribed to the trial, so such costs were not included.^7^

### Cost‐effectiveness analysis

The base‐case analysis comprised a within‐trial incremental CEA conducted from an NHS perspective,^14^ based on the primary outcome of cost per additional live birth at ≥34 weeks of gestation. The trial time horizon was less than a year, so discounting was not applied.

An additional and analogous analysis was performed based on the secondary outcome of neonatal survival at 28 days postpartum. This secondary analysis was reported in terms of cost per additional baby that survived beyond 28 days of birth.

Costs over the trial period were calculated by multiplying the number of resource items used by the corresponding unit cost; these were then added up to obtain the total cost. To account for the inherent skewness of cost data, 95% CIs around mean differences were calculated using the bias‐corrected and accelerated bootstrap method.^17^

Differences in costs and outcomes between the two comparators were calculated using seemingly unrelated estimations.^18^, ^19^ Regression models were used to control for age, body mass index, bleeding quantity and number of previous miscarriages. Incremental cost‐effectiveness ratios (ICERs) were calculated by dividing the difference in mean cost between the trial arms by the difference in the relevant outcomes. All statistical analyses were performed using STATA version 14.^20^ The economic analysis is reported following the Consolidated Health Economic Evaluation Reporting Standards (CHEERS).^21^

### Sensitivity and subgroup analyses

To quantify the uncertainty relating to the assumptions and sampling variations, we conducted sensitivity analyses including (i) one‐way deterministic analyses and (ii) probabilistic sensitivity analyses (PSA). Additionally, (iii) subgroup analyses were carried out to explore the characteristics of patients for whom the intervention might be particularly appropriate.

**I. Deterministic sensitivity analyses**: The range of deterministic sensitivity analyses performed on the input parameters for the base‐case included:

1. **A fixed cost of intervention until 16 weeks**
  In the base‐case analysis, the intervention cost for each woman was calculated using the duration of administration. In practice, progesterone would be provided for the expected treatment period – from confirmation of pregnancy (6–8 weeks) until 16 weeks – hence, we explored the cost impact of progesterone administered for an ideal treatment period.

- **Primary‐care costs**

The base‐case analysis adopted an NHS perspective. To explore the NHS and personal social services perspective, primary‐care costs were included. As there were insufficient primary‐care data, these costs were explored for all participants by imputing missing costs using multiple imputations^22^ by applying chained equations with predictive mean matching across 60 imputations.^23^

- **Unit costs**

The costs of antenatal and postnatal inpatient nights of admission and management termination of pregnancy used in the base‐case analysis were replaced with documented values^8^, ^9^, ^24^ that have been used by other studies.^25^ Furthermore, the impact of removing delivery costs from the base‐case analysis was explored.

**II. Probabilistic sensitivity analysis**: This was conducted for the base‐case and subgroup analyses. PSA comprises non‐parametric bootstrapping (using seemingly unrelated estimates) to resample the joint distribution in the mean cost and outcome difference between the two trial arms.^26^ This generated 5000 paired estimates of incremental costs and live births at ≥34 weeks, and cost‐effectiveness planes were generated using scatterplots.^27^ Cost‐effectiveness acceptability curves (CEACs) were constructed to illustrate the probability that the intervention is cost‐effective at various monetary values that depict decision‐makers’ willingness to pay for an additional live birth.^14^ Detail on the PSA is in the Supplementary material (Appendix [Link], [Link], [Link]).

**III. Subgroup analysis:** Two subgroup analyses were conducted for (i) women with one or more previous miscarriage and (ii) women with three or more previous miscarriages.

## Results

The trial results are reported in detail elsewhere.^7^ Here, we provide the key results for the CEA.

### Participants

A total of 4153 women were recruited and randomised to either the progesterone (*n* = 2079) or placebo (*n* = 2074) arm. Thirty (0.7%) women withdrew from the trial and 85 (2%) women were lost to follow up; hence data were available for 4038 women (2025 in the intervention arm and 2013 in the placebo arm).^7^

### Outcomes

Table 2 presents the results of the trial outcomes required for the economic analysis. For the primary outcome, the proportion of women with live births at ≥34 completed weeks of pregnancy was higher in the progesterone (74.72%) than the placebo (72.48%) arm – an effect difference of approximately 0.022 (2.2%) (95% CI −0.004 to 0.050). There were 1605 versus 1533 babies born alive in the progesterone versus placebo arms, respectively. For the secondary outcome, babies born to 1538 of the 2025 (75.95%) women in the progesterone arm and 1487 of the 2013 (73.87%) women in the placebo arm were still alive at 28 days post‐birth with an effect difference of 0.021 (2.1%) (95% CI −0.005 to 0.048).

**Table 2:** Outcomes across treatment arms

Outcomes	Progesterone		Placebo		Bootstrap difference (95% CI)
	*n/N*%	*n/N*	*n/N*%	*n/N*	
**Primary outcome**					
Live birth beyond 34 weeks	74.72	1513/2025	72.48	1459/2013	0.022 (−0.004 to 0.050)
**Secondary outcome**					
Alive 28 days post‐delivery	75.95	1538/2025	73.87	1487/2013	0.021 (−0.005 to 0.048)

### Resource use and costs

A breakdown of the resource use by the trial arm is provided in the Supplementary material (Table [Link], [Link], [Link])*.* On average, women in the progesterone arm used the intervention for a marginally longer period than those in the placebo arm (50 versus 48 days). For the antenatal period, women allocated to the progesterone arm had, on average, a higher frequency of antenatal and day assessment unit visits, but fewer emergency room visits and hospital admissions than women in the placebo arm. During the postnatal period, women in the progesterone arm used similar services more than those in the placebo arm except for emergency hospital visits, which were similar for both arms. However, women in the placebo arm had more admissions to the HDU and their babies had on average a higher number of admissions to the HDU and the neonatal special care unit than in the intervention.^7^

Table [Link], [Link], [Link] (see Supplementary material) presents the mean costs per woman by trial arm. The average cost of the trial intervention was £204 (95% CI £200 to £207) per pregnancy. Antenatal hospital visits, with a mean cost of £2339 (SD £2672) and £2334 (SD £2665) per woman for the progesterone and placebo arms respectively accounted for the largest proportion of the hospital costs. Mean hospital costs for mother and infant during the trial period were £7655 (SD £9952) in the progesterone arm and £7572 (SD £10,616) in the placebo arm, generating a mean cost difference of £76 (95% CI −£559 to £711). Generally, cost differences between the trial arms were mostly due to the cost of the trial intervention itself (£204, 95% CI £200 to £207), emergency Caesarean section with complications (−£137, 95% CI −£246 to £281) and neonatal high dependency care (−£93, 95% CI −£344 to £159).

### Cost‐effectiveness analysis

The result of the base‐case analysis (Table 3) showed that the intervention group had a mean cost of £7655 per woman. The adjusted (bias‐corrected and accelerated bootstrapped) mean difference was £76 (95% CI –£559 to £711) more than the mean per woman cost of the placebo group (£7572). The progesterone group had a higher proportion of live births at ≥34 weeks, an additional effect of 0.022 (95% CI −0.004 to 0.050), which is equivalent to a gain of two live births per 100 women. The ICER for progesterone relative to placebo was £3305 per additional live birth at ≥34 weeks.

**Table 3:** ICER estimates for the base‐case and secondary analysis

Analysis	Total cost (£) per trial arm	Total effect per trial arm	ICER (progesterone vs placebo – per additional live birth at ≥34 weeks
**Base‐case analysis (Hospital costs for participants and infants)**			
Progesterone	7655	0.747	3305
Placebo	7572	0.725	
Mean difference (95% CI)	76 (−559 to 711)	0.022 (−0.004 to 0.050)	

	Total cost (£) per trial arm	Total effect per trial arm	ICER per additional baby surviving beyond 28 days post‐partum
**Secondary analysis (Hospital costs for participants and infants)**			
Progesterone	7655	0.789	3037
Placebo	7572	0.761	
Mean difference (95% CI)	76 (−559 to 711)	0.021(−0.005 to 0.048)	

For the secondary analysis, which was based on the secondary outcome of the PRISM trial (Table 3) (neonatal survival at 28 days post‐partum), progesterone intervention led to an effect difference of 0.021 (95%CI −0.005 to 0.048), and an ICER of £3037 per additional baby that survived beyond 28 days post‐birth.

### Sensitivity analyses

#### I. Deterministic sensitivity analysis

One‐way deterministic sensitivity analyses required varying costs while keeping the outcome constant. For all scenarios (see Supplementary material, Table [Link], [Link], [Link]), progesterone intervention remained more costly than placebo. The differences in the estimated ICERs were negligible and unlikely to impact decision‐making.

#### II. **Probabilistic sensitivity analysis**

**Figure 1:**
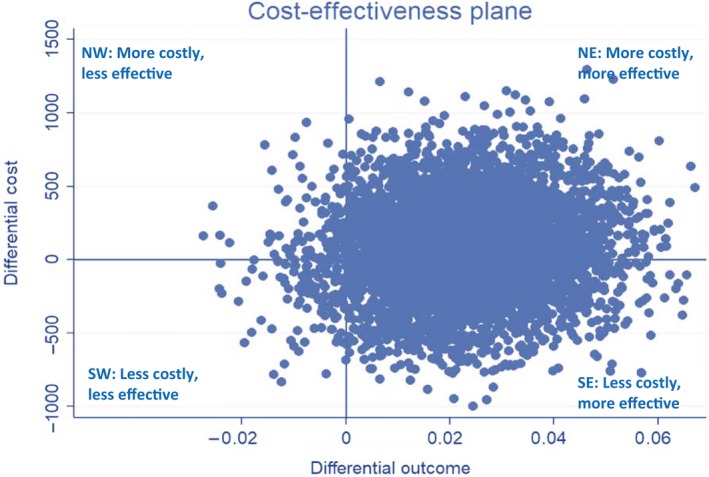
Cost‐effectiveness plane for the base‐case analysis.

The cost‐effectiveness plane for the base‐case analysis is presented in Figure 1. The majority of the scatterplots (depicting paired incremental costs and outcomes) are in the southeast and northeast quadrants. Scatterplots falling in the southeast quadrant represent improved outcome and lower costs, whereas scatterplots in the northeast quadrant represent improved outcome and higher costs. Hence, Figure 1 suggests that progesterone is a more effective intervention. However, it is uncertain whether progesterone is likely to be more costly (northeast) or less costly (southeast) relative to placebo.

**Figure 2:**
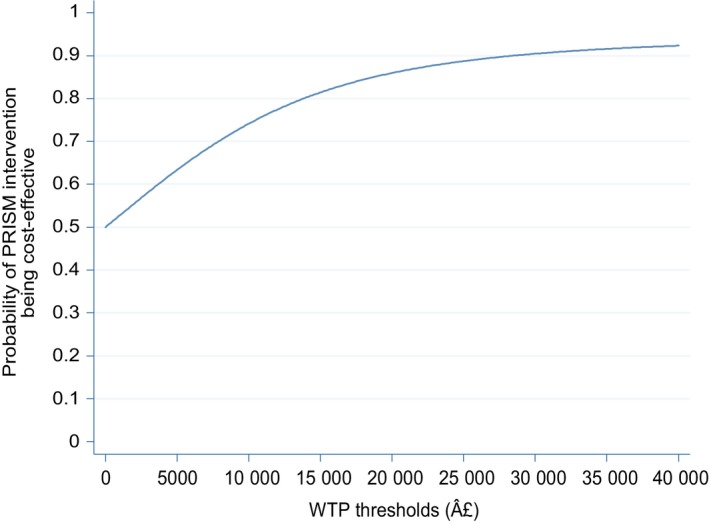
Cost‐effectiveness acceptability curve for the base‐case analysis.

The CEAC (Figure 2) shows the probability of progesterone being cost‐effective at various values of decision‐makers’ willingness to pay (WTP) per additional live birth. For thresholds of WTP per additional live birth greater than £15,000, there is >80% probability that progesterone is cost‐effective. The probability of cost‐effectiveness steadily increases and exceeds 90% for WTP thresholds greater than £23,000.

#### III. **Subgroup analysis**

The subgroup analysis (Table 4) conducted on women with at least one previous miscarriage found the trial intervention to be less costly, with a cost saving of £322 (95% CI −£1318 to £673) but more effective with an additional gain of five live births per 100 women (0.055, 95% CI 0.014–0.096).

**Table 4:** Results of the subgroup analyses

Analysis	Total cost (£) per trial arm	Total effect per trial arm	ICER (progesterone vs placebo – per additional live birth at ≥34 weeks
**Subgroup analysis (Hospital costs for participants with at least one previous miscarriage)**			
Progesterone	7705	0.754	Progesterone dominant
Placebo	8072	0.699	
Mean difference (95% CI)	−322 (−1318 to 673)	0.055 (0.014 to 0.096)	
**Subgroup analysis (Hospital costs for participants with three or more previous miscarriages)**			
Progesterone	9304	0.715	11 606
Placebo	7803	0.574	
Mean difference (95% CI)	1754 (−1041 to 4550)	0.151 (0.042 to 0.260)	

The cost‐effectiveness plane (see Supplementary material, Figure [Link], [Link], [Link]A), with the majority of dots in the southeast quadrant, suggests that progesterone is less costly and more effective than placebo. The CEAC (see Supplementary material, Figure [Link], [Link], [Link]B) shows that for WTP thresholds greater than £20,000, the probability of progesterone being cost‐effective is >90%. The cost‐effectiveness plane and CEAC for this subgroup suggest that progesterone is likely to be considered a dominant intervention compared with placebo.

For women with three or more previous miscarriages, progesterone intervention was both more costly (with a cost difference of £1754, 95% CI –£1041 to £4550) and more effective (with an additional gain of 15 live births per 100 women). The ICER was £11,606 per additional live birth at ≥34 weeks. The cost‐effectiveness plane for this subgroup (see Supplementary material, Figure [Link], [Link], [Link]A) shows a majority of the dots in the northeast quadrant, which represents improved outcome but higher costs. The CEAC (see Supplementary material, Figure [Link], [Link], [Link]B) also shows the probability of progesterone being cost‐effective is >90% for WTP thresholds greater than £20,000. The differences in cost compared with both the base‐case and the subgroup of women with at least one previous miscarriage, were mostly driven by higher neonatal care costs incurred by women with three or more previous miscarriages (see Supplementary material, Figure [Link], [Link], [Link]). Although the trial intervention led to more live births for this group, some of these babies required neonatal intensive care, HDU or special care, thereby generating a higher cost difference than the base‐case and the other subgroup (see Supplementary material, Figure [Link], [Link], [Link]).

## Discussion

### Main findings

This study assessed the cost‐effectiveness of progesterone compared with placebo in avoiding miscarriage and achieving a live birth at ≥34 weeks of pregnancy in women who presented with bleeding in early pregnancy. The results suggest that progesterone treatment is more costly with an average cost per participant of £7655 compared with £7572 for placebo. The difference in costs (£76, 95% CI −£559 to £711) was mainly attributable to the cost of progesterone (£204). Progesterone resulted in an additional effect of 0.022 (95% CI −0.004 to 0.050).

Although there is statistical uncertainty around the clinical data points, when using the approaches required by the guidelines for economics analysis,^21^, ^28^ in which the uncertainty must be estimated using confidence intervals around the point estimates, the economic analysis suggests that progesterone has a small positive effect compared with placebo. Consequently, the base‐case economic analysis suggests that progesterone intervention is slightly more costly and slightly more effective than placebo and the estimated ICER is £3305 per additional live birth at ≥34 weeks.

In the analogous analysis based on the secondary outcome (Table 2), the intervention was slightly more effective, with an estimated gain of two neonates (0.021, 95% CI −0.005 to 0.048) surviving beyond 28 days post‐partum per 100 women. The ICER was £3037 per additional baby surviving beyond 28 days post‐birth.

The subgroups analyses show a clear result in favour of progesterone use for women with bleeding and a previous history of miscarriage. For the subgroup of women with at least one previous miscarriage, the analysis shows that the intervention with progesterone would be less costly and more effective and suggests that progesterone is a dominant intervention for this group. For the subgroup of women with three or more previous miscarriages, there is an increase in the ICER compared with the base‐case although it is still likely to be considered favourable.

It is notable that for the subgroup analyses of women with three or more previous miscarriages, women in the progesterone arm on average incurred more neonatal care costs than women in the placebo arm. A tentative implication of this is the success of progesterone, averting miscarriage leading to more live births requiring neonatal intensive care. However, the main (base‐case) finding showed that overall, women in the placebo arm on average used more neonatal care resources than those in the progesterone arm.

### Strengths and limitations

A strength of this economic evaluation is that it is based on the largest multi‐centre randomised clinical trial (over 4000 participants), which explored whether progesterone is clinically effective in preventing miscarriage in women with early pregnancy bleeding. The data were prospectively collected at different points in the trial. Unit costs were obtained from standard and recognised sources. The cost‐effectiveness results also benefited from the robustness of the main analyses and sensitivity analyses.^7^ Although data on primary‐care services were available for <10% of the participants this was accounted for by imputing missing costs.

A potential limitation of this study is the confusion that might arise given that the reported clinical results for the base‐case suggested the additional effect of progesterone was not statistically significantly different from the placebo,^7^ whereas the health economics analysis suggests that progesterone given to women who have threatened miscarriage in early pregnancy is likely to be cost‐effective. This contrasting interpretation of the results relates to a requirement in the recommendations for health economics analysis to estimate and quantify the uncertainty around the clinical end‐points (based on appropriate distributions applied to the confidence intervals surrounding the point estimate and using probabilistic sensitivity analysis).^21^, ^28^ This recommended and widely endorsed approach to estimating the uncertainty is recognised as potentially challenging and has been widely debated and explained elsewhere.^14^, ^28^, ^29^ This approach, advocated in health economics guidelines^14^, ^21^ has very recently received attention and support from elsewhere.^30^, ^31^

It was beyond the scope of and timeline of this study to explore the wider societal costs to the participants, given the potential impact on society of fewer miscarriages but the wider societal perspective is not anticipated to oppose the direction of the results reported here.

### Interpretation

Whether progesterone would be supported in resource allocation decisions depends on the amount that society is willing to pay to increase the chances of an additional live birth at ≥34 weeks of gestation. There is currently no threshold values assigned to an additional live birth.^25^, ^32^ The base‐case analysis (Figure 2) suggests that the probability of progesterone being cost‐effective is >50% for almost all values represented in the CEAC. The subgroup analysis for at least one miscarriage (see Supplementary material, Figure [Link], [Link], [Link]B) is more convincing. Given the distress to women and families associated with miscarriage, and the subsequent resources that might be associated with counselling and close antenatal attention in the subsequent pregnancies of women who experience miscarriage,^33^ the costs of which were beyond the remit of the current study, progesterone is likely to be considered good value for money in preventing miscarriage.

### Comparison with the literature

This is the first UK research to examine the cost‐effectiveness of progesterone in achieving a live birth beyond 34 weeks in women with threatened miscarriage. A similar UK study explored the cost‐effectiveness of progesterone in preventing miscarriages in women with a history of unexplained recurrent miscarriages leading to a live birth beyond 24 weeks.^25^ The authors found that the total mean cost of the intervention was higher in the progesterone arm than in the placebo arm by £332 and an ICER of £18,053 per additional live birth beyond 24 weeks for the base‐case analysis with a 50% probability of being cost‐effective at this value.^25^

## Conclusion

Currently, in the UK, progesterone is not routinely given to women who are at high risk for a miscarriage.^6^ The results of the CEA reported here suggest that progesterone is likely to be cost‐effective for all women at risk, but particularly for women with one or more previous miscarriages who present with bleeding in early pregnancy. This analysis lends credibility to the belief that progesterone should be given to such women^7^ on cost‐effectiveness grounds.

### Disclosure of interests

There are no conflicts of interests to disclose. Completed disclosure of interests forms are available to view online as supporting information.

### Contribution to authorship

AC, AD, TER, HH, JPD, WCD, AKE, JN and SQ were co‐applicants and contributed to the design of the study. AC and AD were involved with the implementation and oversight of the trial. AC was the Chief Investigator and AD was the trial manager. CO, IG and TER were responsible for the Health Economics. CO was the principal HE researcher and received advice from IG and TER. TER was the principal economic supervisor. LJM and VC were responsible for the analyses of the trial data. LJM was the senior statistician and VC was the trial statistician. HH, IDG, AE and KK were involved with recruitment. KH, JR, AA, KB, CB, MC, SD, TH,FI, JJ, ML, PM, NN, CO, SR, AS, MU, NV, LW, CW, AWH, DJ, JPD, AKE, KK, IG, RB, JB WCD, AE, JN and SQ were Principal Investigators for the trial. CO was responsible for the first draft of this manuscript; IG, TER, AD, AC, VC, LJM, HH and IDG all contributed to the drafting of the manuscript. All individuals contributed to the editing and revision of the manuscript.

### Details of ethics approval

The trial had a favourable ethical opinion from the National Research Ethics Service (NRES) Committee South Central (Oxford C), 26/11/2014, ref: 14‐SC‐1345.

### Funding

The study was funded by the UK NIHR Health Technology Assessment programme (project number HTA 12/167/26). ISRCTN: 14163439. The views and opinions expressed by authors in this publication are those of the authors and do not necessarily reflect those of the NHS, the NIHR, the Medical Research Council, the Central Commissioning Facility, the NIHR Evaluation, Trials and Studies Coordinating Centre, the Health Technology Assessment Programme, or the Department of Health.

### Acknowledgements

We thank all the women who participated in this study; the following investigators for supervising recruitment and randomisation at the study centres: Mr Samson Agwu, Mrs Rita Arya, Miss Miriam Baumgarten, Dr Catey Bass, Miss Sumita Bhuiya, Prof Tom Bourne, Mr James Clark, Mr Samual Eckford, Mr Zeiad El‐Gizawy, Mrs Joanne Fletcher, Miss Preeti Gandhi, Dr Mary Gbegaje, Dr Ingrid Granne, Mr Mamdough Guirguis, Dr Pratima Gupta, Dr Hadi Haerizadeh, Dr Laura Hipple, Mr Piotr Lesny, Miss Hema Nosib, Mr Jonathan Pepper, Mr Jag Samra, Ms Jayne Shillito, Dr Rekha Shrestha, Dr Jayasree Srinivasan, Dr Ayman Swidan and Prof Derek Tuffnell; all the PRISM research nurses who assisted in the collection of data and the Early Pregnancy Unit staff who supported the trial; Leanne Beeson, Mary Nulty and Louisa Edwards for their support in managing and coordinating the trial; Prof Siladitya Bhattacharya for chairing the trial steering committee; Prof Andrew Shennan for chairing the data and safety monitoring committee; Dr Javier Zamora and Dr Willem Ankum for participating in the data monitoring committee; Dr Pelham Barton who gave health economics analysis advice; and all those not otherwise mentioned above who have contributed to the PRISM study.

## Supporting information


**Figure S1**. Cost‐effectiveness plane and cost‐effectiveness acceptability curve (CEAC) for the subgroup analysis of women with at least one previous miscarriage.
**Figure S2**. Cost‐effectiveness plane and cost‐effectiveness acceptability curve for the subgroup analysis of women with three or more previous miscarriages.
**Figure S3**. (a) Cost breakdown for women in progesterone arm – the base‐case versus women with three or more miscarriages. (b) Cost breakdown for women in progesterone arm – women with at least one miscarriage versus women with three or more miscarriages.

**Table S1**. Mean resource use across treatment arms.
**Table S2**. Disaggregated costs by trial arms (prices in 2017/18 pounds sterling).
**Table S3**. Sensitivity analyses.

**Appendix S1**. Exclusion criteria.
**Appendix S2**. Probabilistic sensitivity analysis.

## References

[1] Wilcox AJ . Fertility and Pregnancy: An Epidemiologic Perspective. Oxford: Oxford University Press; 2010.

[2] Wilson R , Jenkins C , Miller H , McInnes IB , Moore J , McLean MA , et al. Abnormal cytokine levels in non‐pregnant women with a history of recurrent miscarriage. Eur J Obstet Gynecol Reprod Biol 2004;115:51–4. 15223165 10.1016/j.ejogrb.2003.11.029

[3] Jurkovic D , Overton C , Bender‐Atik R . Diagnosis and management of first‐trimester miscarriage. BMJ 2013;346:f3676. 23783355 10.1136/bmj.f3676

[4] Regan L , Rai R . Epidemiology and the medical causes of miscarriage. Best Pract Res Clin Obstet Gynaecol 2000;14:839–54. 10.1053/beog.2000.0123 11023804

[5] Kong G , Chung T , Lai B , Lok I . Gender comparison of psychological reaction after miscarriage—a 1‐year longitudinal study. BJOG 2010;117:1211–19. 20618319 10.1111/j.1471-0528.2010.02653.x

[6] Newbatt E , Beckles Z , Ullman R , Lumsden MA . Ectopic pregnancy and miscarriage: summary of NICE guidance. BMJ 2012;345:e8136. 23236034 10.1136/bmj.e8136

[7] Coomarasamy A , Devall AJ , Cheed V , Harb H , Middleton LJ , Gallos ID , et al. A randomized trial of progesterone in women with bleeding in early pregnancy. N Engl J Med 2019;380:1815–24. 31067371 10.1056/NEJMoa1813730

[8] National Collaborating Centre for Women's and Children's Health . Ectopic Pregnancy and Miscarriage: Diagnosis and Initial Management in Early Pregnancy of Ectopic Pregnancy and Miscarriage. London: National Collaborating Centre for Women's and Children's Health; 2012.

[9] Reference Cost Collection: National Schedule of Reference Costs, 2016‐17 –NHS trusts and NHS foundation trusts [Internet]. 2017 [https://improvement.nhs.uk/documents/3479/201617_ReferenceCostData.zip]. Accessed 29 June 2018.

[10] Curtis LA , Burns A . Unit Costs of Health and Social Care 2017. Canterbury: Personal Social Services Research Unit, University of Kent; 2017.

[11] NHS Reference Costs 2013/14 [Internet]. 2014 [https://www.gov.uk/government/publications/nhs-reference-costs-2013-to-2014/03a_2013-14_National_Schedule_-_CF-NET_updated]. Accessed 28 June 2018.

[12] Curtis L , Burns A . Unit Costs of Health and Social Care 2015. Canterbury: Personal Social Services Research Unit, University of Kent; 2015.

[13] Netten A , Curtis L . Unit Costs of Health and Social Care 2002. Canterbury: Personal Social Services Research Unit, University of Kent, 2002.

[14] NICE . Guide to the Methods of Technology Appraisal 2013. London: National Institute for Health and Care Excellence; 2013. 27905712

[15] Committee JF . British National Formulary (online). London: BMJ Group and Pharmaceutical Press; 2017.

[16] Intensive Care Society . Levels of Critical Care for Adult Patients. London: Intensive Care Society; 2009.

[17] Barber JA , Thompson SG . Analysis of cost data in randomized trials: an application of the non‐parametric bootstrap. Stat Med 2000;19:3219–36. 11113956 10.1002/1097-0258(20001215)19:23<3219::aid-sim623>3.0.co;2-p

[18] Zellner A , Huang DS . Further properties of efficient estimators for seemingly unrelated regression equations. Int Econ Rev 1962;3:300–13.

[19] Moon HR , Perron B . Seemingly unrelated regressions. The New Palgrave Dictionary of Economics. 2006;1(9).

[20] Stata Corp LP . Stata Statistical Software Release 15. College Station: Stata Press Publication; 2017.

[21] Husereau D , Drummond M , Petrou S , Carswell C , Moher D , Greenberg D , et al. Consolidated health economic evaluation reporting standards (CHEERS)—explanation and elaboration: a report of the ISPOR health economic evaluation publication guidelines good reporting practices task force. Value in Health 2013;16:231–50. 23538175 10.1016/j.jval.2013.02.002

[22] Rubin DB . Multiple Imputation for Nonresponse in Surveys. Hoboken: John Wiley & Sons; 2004.

[23] White IR , Royston P , Wood AM . Multiple imputation using chained equations: issues and guidance for practice. Stat Med 2011;30:377–99. 21225900 10.1002/sim.4067

[24] Petrou S , Trinder J , Brocklehurst P , Smith L . Economic evaluation of alternative management methods of first‐trimester miscarriage based on results from the MIST trial. BJOG 2006;113:879–89. 16827823 10.1111/j.1471-0528.2006.00998.x

[25] Coomarasamy A , Williams H , Truchanowicz E , Seed PT , Small R , Quenby S , et al. A randomized trial of progesterone in women with recurrent miscarriages. N Engl J Med 2015;373:2141–8. 26605928 10.1056/NEJMoa1504927

[26] Glick HA , Briggs AH , Polsky D . Quantifying stochastic uncertainty and presenting results of cost‐effectiveness analyses. Exp Rev Pharmacoecon Outcomes Res 2001;1:25–36. 10.1586/14737167.1.1.25 19807505

[27] Black WC . The CE plane: a graphic representation of cost‐effectiveness. Med Decis Making 1990;10:212–4. 2115096 10.1177/0272989X9001000308

[28] Whitehurst DG , Bryan S . Trial‐based clinical and economic analyses: the unhelpful quest for conformity. Trials 2013;14:421. 24308301 10.1186/1745-6215-14-421 PMC4233716

[29] Bayarri MJ , Berger JO . The interplay of Bayesian and frequentist analysis. Stat Sci 2004;19:58–80.

[30] Amrhein V , Greenland S , McShane B . Scientists rise up against statistical significance. Nature 2019;567:305–7. 30894741 10.1038/d41586-019-00857-9

[31] Betensky R , Newberger N . New guidelines for statistical reporting. N Engl J Med 2019;381:1597. 10.1056/NEJMc1911817 31618557

[32] Simon J , Petrou S , Gray A . The valuation of prenatal life in economic evaluations of perinatal interventions. Health Econ 2009;18:487–94. 18615854 10.1002/hec.1375

[33] Ogwulu CB , Jackson LJ , Heazell AE , Roberts TE . Exploring the intangible economic costs of stillbirth. BMC Pregnancy Childbirth 2015;15:188. 26323522 10.1186/s12884-015-0617-x PMC4556317

