# Supplementary material for: The cost‐effectiveness of progesterone in preventing miscarriages in women with early pregnancy bleeding: an economic evaluation based on the PRISM trial
Source: BJOG. 2020 Jan 30;127(6):757–67. doi: 10.1111/1471-0528.16068 (PMC7187468; doi:10.1111/1471-0528.16068)
Supplement: Supplementary file 1 — Figure S1. Cost‐effectiveness plane and cost‐effectiveness acceptability curve (CEAC) for the subgroup analysis of women with at least one previous miscarriage. Figure S2. Cost‐effectiveness plane and cost‐effectiveness acceptability curve for the subgroup analysis of women with three or more previous miscarriages. Figure S3. (a) Cost breakdown for women in progesterone arm – the base‐case versus women with three or more miscarriages. (b) Cost breakdown for women in progesterone arm – women with at least one miscarriage versus women with three or more miscarriages. [file BJO-127-757-s001.pdf]

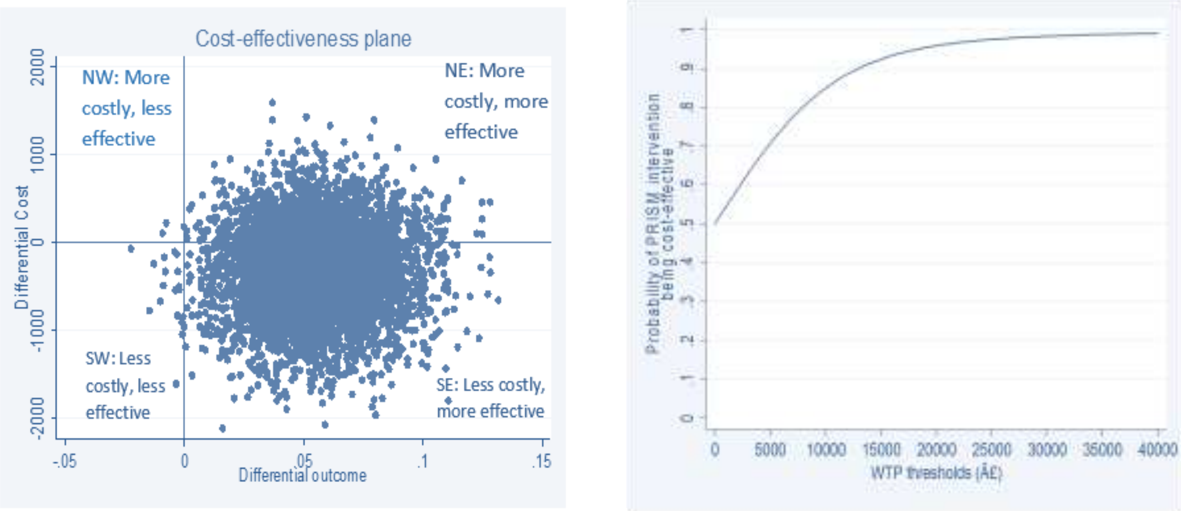

Figure S1. Cost-effectiveness plane and Cost-effectiveness acceptability curve (CEAC) for the subgroup analysis of women with ≥1 previous miscarriage.

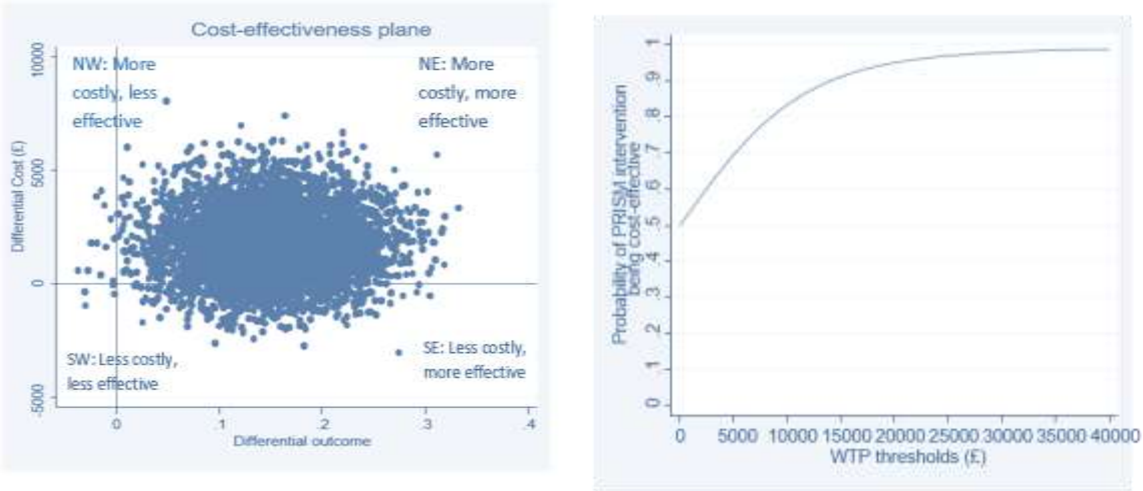

Figure S2. Cost-effectiveness plane and cost-effectiveness acceptability curve for the subgroup analysis of women with ≥3 previous miscarriages.

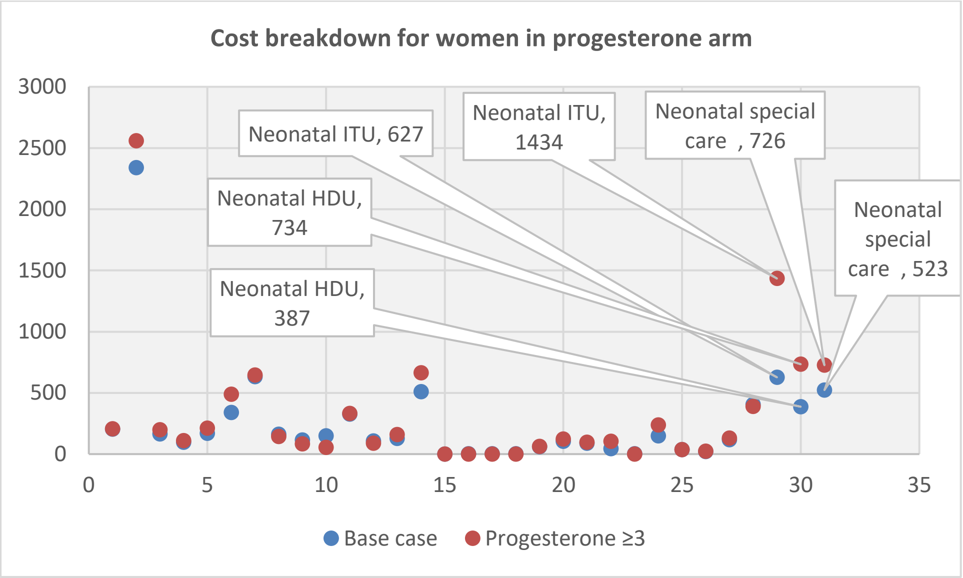

Figure S3a. Cost breakdown for women in progesterone arm - the base case versus women with 3 or more miscarriages.
(HDU - High dependency unit, ITU - Intensive unit)

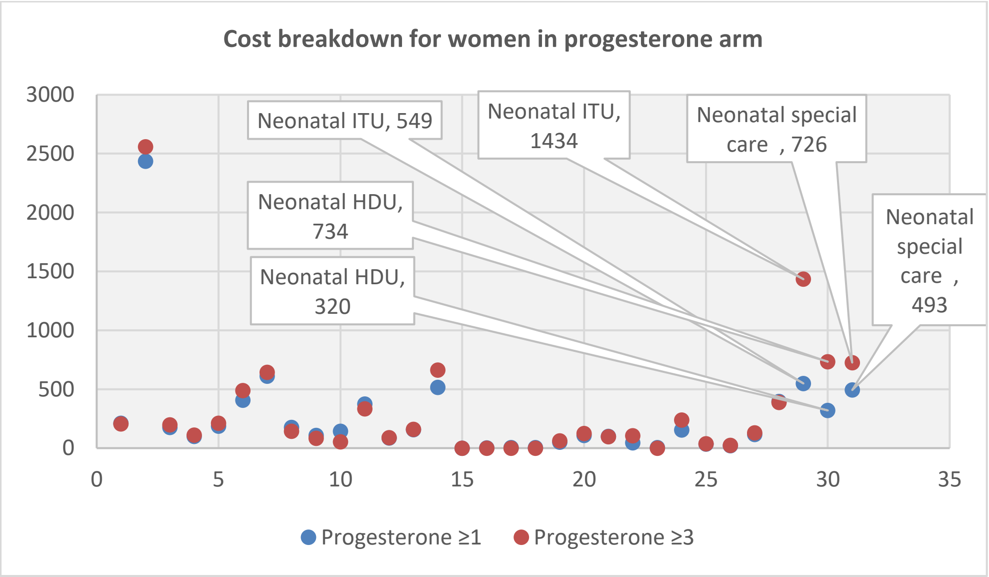

Figure S3b. Cost breakdown for women in progesterone arm - Women with ≥1 miscarriage versus women with ≥3 miscarriages.
(HDU - High dependency unit, ITU - Intensive unit)
