# Supplementary material for: The cost‐effectiveness of progesterone in preventing miscarriages in women with early pregnancy bleeding: an economic evaluation based on the PRISM trial
Source: BJOG. 2020 Jan 30;127(6):757–67. doi: 10.1111/1471-0528.16068 (PMC7187468; doi:10.1111/1471-0528.16068)
Supplement: Supplementary file 2 — Table S1. Mean resource use across treatment arms. Table S2. Disaggregated costs by trial arms (prices in 2017/18 pounds sterling). Table S3. Sensitivity analyses. [file BJO-127-757-s002.pdf]

Table S1. Mean resource use across treatment arms

Resource items	Progesterone		Placebo		Bootstrap Adjusted mean difference (95% CI)
	N	Mean (SD)	N	Mean (SD)	
**Days receiving progesterone or placebo**	2023	50.40 (21.11)	2009	48.43 (22.01)	2.02 (0.72 to 3.31)
**Antenatal Period**					
Antenatal hospital visit	2020	5.00 (5.71)	2005	4.99 (5.69)	0.01 (-0.34 to 0.36)
Day assessment unit	2020	1.32 (2.50)	2005	1.26 (2.38)	0.06 (-0.09 to 0.21)
Emergency visit	2020	0.81 (1.51)	2005	0.89 (1.59)	-0.07 (-0.16 to 0.02)
Inpatient admission (<24 hours)	2020	0.57 (1.02)	2005	0.59 (1.08)	-0.03 (-0.09 to 0.04)
Nights of admission (duration, days)	2020	0.86 (2.55)	2005	0.96 (2.99)	-0.09 (-0.26 to 0.08)
**Mode of delivery**					
Unassisted vaginal delivery (without cc)	695	0.34 (0.48)	673	0.33 (0.47)	0.009 (-0.02 to 0.04)
Unassisted vaginal delivery (with cc)	150	0.07 (0.26)	122	0.06 (0.24)	0.014 (0.00 to 0.03)
Instrumental vaginal delivery (without cc)	101	0.05 (0.22)	93	0.05 (0.21)	0.004 (-0.01 to 0.02)
Instrumental vaginal delivery (with cc)	123	0.06 (0.24)	107	0.05 (0.22)	0.008 (-0.007 to 0.02)
Elective caesarean section (without cc)	204	0.10 (0.30)	172	0.09 (0.28)	0.015 (-0.004 to 0.03)
Elective caesarean section (with cc)	53	0.03 (0.16)	52	0.03 (0.16)	0.000 (-0.01 to 0.01)
Emergency caesarean section (without cc)	59	0.03 (0.17)	56	0.03 (0.16)	0.001 (-0.009 to 0.01)
Emergency caesarean section (with cc)	182	0.09 (0.29)	230	0.11 (0.32)	-0.024 (-0.043 to -0.006)
Vaginal breech delivery (without cc)	1	0.00 (0.02)	3	0.00 (0.03)	0.000 (-0.002 to 0.001)
Vaginal breech delivery (with cc)	3	0.00 (0.04)	5	0.00 (0.05)	-0.001 (-0.004 to 0.002)
Other (without cc)	3	0.00 (0.04)	3	0.00 (0.04)	0.00 (-0.002 to 0.002)
Other (with cc)	3	0.00 (0.04)	2	0.00 (0.03)	0.00 (-0.002 to 0.003)
**Miscarriage management**					
Spontaneous resolution	197	0.10 (0.30)	243	0.12 (0.33)	-0.007 (-0.022 to 0.007)
Surgical	112	0.06 (0.23)	125	0.06 (0.24)	-0.023 (-0.043 to -0.004)
Medical	97	0.05 (0.21)	91	0.05 (0.21)	0.003 (-0.001 to 0.015)
**Postnatal period**					
Admission to HDU (level 2 care)	2006	0.05 (0.30)	1991	0.06 (0.46)	-0.01 (-0.035 to 0.012)
Admission to ITU (level 3 care)	2006	0.00 (0.04)	1991	0.00 (0.03)	0.00 (-0.001 to 0.003)
Hospital visit	1984	1.03 (2.98)	1963	1.02 (2.88)	0.01 (-0.17 to 0.20)
Day assessment unit	1984	0.30 (1.35)	1963	0.27 (1.13)	0.03 (-0.04 to 0.11)
Emergency visit	1984	0.22 (0.84)	1963	0.22 (0.85)	-0.00 (-0.06 to 0.05)
Inpatient admission	1984	0.40 (0.59)	1963	0.37 (0.60)	0.03 (-0.007 to 0.066)

Nights of inpatient admission	1984	1.03 (1.99)	1963	0.96 (1.76)	0.07 (-0.04 to 0.19)
**Neonatal period**					
Neonatal intensive care	1565	0.48 (4.76)	1502	0.48 (4.57)	-0.00 (-0.34 to 0.32)
Neonatal high dependency care	1565	0.42 (4.02)	1502	0.52 (3.90)	-0.10 (-0.38 to 0.18)
Neonatal special care	1565	1.02 (5.07)	1503	1.16 (4.95)	-0.15 (-0.50 to 0.21)
***Primary care services**					
GP contact	133	0.64 (1.21)	133	0.77 (1.25)	-0.12 (-0.40 to 0.15)
Practice/Community Midwife contact	132	2.69 (3.77)	133	1.95 (3.09)	0.79 (-0.01 to 1.60)
Practice nurse contact	136	0.18 (0.61)	136	0.16 (0.47)	0.02 (-0.12 to 0.15)
Psychologist (or counsellor) visit	136	0.18 (1.05)	136	0.05 (0.52)	0.15 (-0.07 to 0.37)
Health visitor visit	133	0.39 (0.79)	136	0.29 (0.69)	0.10 (-0.08 to 0.28)
Social worker visit (Adult)	136	0.04 (0.51)	136	0.00 (0.00)	0.04 (-0.02 to 0.11)
Other community services	134	0.16 (0.78)	133	0.13 (0.45)	0.02 (-0.13 to 0.17)

The primary care mean values were calculated for only participants with complete primary care data

cc, complications; HDU high dependency unit; ITU, intensive care unit; GP, general practitioner

Table S2. Disaggregated costs by trial arms (prices in £2017-18)

Resource items	Progesterone (n=2025)	Placebo (n=2013)	Bootstrap mean cost difference (95% CI)
	Mean (SD)	Mean (SD)	
**Intervention**	204 (84)	0 (0)	204 (200 to 207)
**Antenatal period**			
Hospital visit	2339 (2672)	2334 (2665)	4 (-159 to 166)
Day assessment unit	164 (312)	158 (297)	8 (-11 to 26)
Emergency visit	96 (179)	105 (188)	-9 (-20 to 2)
Inpatient admission	171 (309)	180 (327)	-8 (-28 to 12)
Nights of admission	341 (1006)	378 (1182)	-36 (102 to 29)
**Delivery mode**			
Unassisted vaginal delivery (without cc)	632 (874)	615 (868)	18 (-36 to 71)
Unassisted vaginal delivery (with cc)	162 (573)	132 (513)	30 (-3 to 63)
Instrumental vaginal delivery (without cc)	115 (501)	106 (483)	8 (-23 to 39)
Instrumental vaginal delivery (with cc)	149 (584)	130 (549)	19 (-15 to 53)
Elective caesarean section (without cc)	328 (981)	278 (911)	48 (-11 to 108)
Elective caesarean section (with cc)	107 (651)	105 (647)	1 (-40 to 41)
Emergency caesarean section (without cc)	128 (737)	122 (720)	5 (-38 to 48)
Emergency caesarean section (with cc)	510 (1624)	649 (1807)	-137 (-246 to -28)
Vaginal breech delivery (without cc)	1 (41)	2 (58)	-1 (-4 to 2)
Vaginal breech delivery (with cc)	3 (84)	5 (109)	-2 (-8 to 4)
Other (without cc)	3 (71)	3 (71)	-0 (-4 to 4)
Other (with cc)	3 (84)	2 (69)	1(-4 to 6)
**Miscarriage management**			
Spontaneous resolution	60 (183)	75 (202)	-14 (-27 to -2)
Surgical	104 (430)	117 (454)	-13 (-40 to 13)
Medical	90 (402)	85 (391)	5 (-20 to 30)
**Postnatal services**			
Admission to HDU (level 2 care)	44 (288)	55 (448)	-11 (-34 to 12)
Admission to ITU (level 3 care)	3 (71)	2 (50)	2 (-2 to 6)
Hospital visit	150 (431)	148 (417)	2 (-23 to 27)
Day assessment unit	38 (169)	34 (141)	4 (-5 to 14)
Emergency visit	21 (82)	22 (83)	-0 (-6 to 5)
Inpatient admission	118 (175)	110 (180)	9 (-3 to 20)

Night of inpatient admission	406 (786)	378 (694)	29 (-19 to 77)
**Neonatal services**			
Neonatal intensive care	627 (6275)	634 (6017)	-10 (-442 to 421)
Neonatal high dependency care	387 (3670)	477 (3560)	-93 (-344 to 159)
Neonatal special care	523 (2605)	595 (2543)	-76 (-260 to 109)
**Mean Total costs**			
**Hospital-related**	7452 (9935)	7572 (10616)	-127 (-759 to 505)
**Hospital-related plus intervention**	7655 (9952)	7572 (10616)	76 (-559 to 711)
***Primary care services**			
GP contacts	25 (47)	30 (49)	-5 (-16 to 6)
Practice/Community Midwife	81 (113)	57 (93)	24 (-1 to 49)
Practice nurse contacts	2 (6)	2 (5)	0.18 (-1 to 1)
Psychologist (or counsellor) visits	4 (20)	1 (10)	3 (-1 to 7)
Health visitor visits	9 (17)	6 (15)	2 (-2 to 6)
Social worker visits (Adult)	1 (11)	-	1 (-0 to 2)
Number of other community services	3 (16)	3 (9)	0.36 (-3 to 3)

**The primary care mean values were calculated for only participants with complete primary care data*

Table S3. Sensitivity Analyses

Sensitivity analyses	Total costs (£)		Adjusted mean Cost difference (£)	ICER (£) (Per additional live birth at ≥ 34 weeks)
	Progesterone	Placebo		
*Fixed progesterone cost until 16 weeks	7694	7572	115 (-506 to 735)	4977
Imputation of primary care costs	7753	7666	100 (-532 to 731)	4321
**Varying cost of inpatient nights of admission	7498	7413	77 (-536 to 691)	3356
***Varying cost of miscarriage management	7635	7552	76 (-546 to 697)	3282
Removing delivery costs	5515	5421	86 (-500 to 672)	3743

* If it is assumed that all women commenced the intervention at approximately 7.4 weeks (52 days) (based on trial data) with no miscarriage, until 16 weeks (112 days) of pregnancy at which the intervention was stopped, this is equivalent to 60 days and a cost of £238 [2 +(59 × 4].

**The costs of antenatal/postnatal inpatient night of admission were replaced with the cost of excess bed days (£311).

***Miscarriage management costs were replaced with values (£1522 and £1827 for medical and surgical management respectively) from the NICE guideline on miscarriage [8, 24]
