# Supplementary material for: The cost‐effectiveness of progesterone in preventing miscarriages in women with early pregnancy bleeding: an economic evaluation based on the PRISM trial
Source: BJOG. 2020 Jan 30;127(6):757–67. doi: 10.1111/1471-0528.16068 (PMC7187468; doi:10.1111/1471-0528.16068)
Supplement: Supplementary file 3 — Appendix S1. Exclusion criteria. Appendix S2. Probabilistic sensitivity analysis. [file BJO-127-757-s003.pdf]

Appendix S1. Exclusion Criteria

Women were not included in the trial if at the time of presentation:

- The fetal crown-rump length was 7 mm or longer with no visible heartbeat;
- If the gestational sac was a mean of 25 mm or greater in diameter with no visible fetal pole on ultrasonography;
- If they had evidence of ectopic pregnancy; if they had life-threatening bleeding;
- If they had current or recent use of progesterone supplementation;
- If they had contraindications to progesterone therapy (i.e., a history of liver tumours; current genital or breast cancer, severe arterial disease, or acute porphyria; or a history during pregnancy of idiopathic jaundice, severe pruritus, or pemphigoid gestations); or
- If they were participating in any other blinded, placebo-controlled trials of medicinal products in pregnancy.

Appendix S2. Probabilistic Sensitivity Analysis

The approach taken in the probabilistic sensitivity analysis is that all important variables relating to costs and clinical outcomes are given a distribution that describes the uncertainty surrounding the mean. The distributions are simulated 5000 times. Each time, random numbers are drawn from the appropriate distributions. After each simulation, the incremental costs and effects are plotted in a cost-effectiveness plane which comprises 4 quadrants: north-east (NE), north-west (NW), south-east (SE) and south-west (SW). The scatterplot that is produced represents the simulations. If dots from the scatterplot are in the NE quadrant this indicates the intervention is more costly and more effective compared to the comparator. Dots in the SE quadrant indicate that intervention is less costly and more effective than the comparator. Based on these simulations, the probability that the intervention would be cost-effective is presented. This is the standard approach for health economics following accepted guidelines (CHEERS)[21] and is a presentation of results of cost-effectiveness studies which would be required by decision-makers such as NICE.[14]
